# Comparison of Sample Preparation and Determination of 60 Veterinary Drug Residues in Flatfish Using Liquid Chromatography-Tandem Mass Spectrometry

**DOI:** 10.3390/molecules25051206

**Published:** 2020-03-07

**Authors:** Joohye Kim, Hyunjin Park, Hui-Seung Kang, Byung-Hoon Cho, Jae-Ho Oh

**Affiliations:** Pesticide and Veterinary Drug Residues Division, National Institute of Food and Drug Safety Evaluation, Osong, Chungcheongbuk-do 28165, Korea; joohye@korea.kr (J.K.); hj77park@korea.kr (H.P.); chobh02@korea.kr (B.-H.C.); chopin68@korea.kr (J.-H.O.)

**Keywords:** veterinary medicine, multi-residue, analytical method, aquatic animal, LC-MS/MS

## Abstract

This study was performed to optimize the analytical method for multi-residues of 60 compounds in flatfish samples. Three sample preparation methods were tested to identify the optimal recovery conditions for target analytes. As a result, 10 mL of water/acetonitrile (1:4, *v*/*v*) was used to extract analytes from fish samples. For purification, C_18_ and 10 mL of acetonitrile saturated hexane were used to treat the samples. After evaporation and reconstitution, the fish samples were analyzed by ultra-performance liquid chromatography-tandem mass spectrometry. The proposed method was validated according to the CODEX guidelines (CAC/GL-71). Our results showed the recoveries of 73.2–115% and coefficients of variation of 1.6–22.1%. The limit of quantification was 0.0005–0.005 mg/kg in the fishery products. In analysis of real samples, no samples exceeded the limit of quantification. This analytical method can be used for multi-residue screening and confirmation of the residues of veterinary drugs in fishery products.

## 1. Introduction

The aquaculture industry continues to expand, providing fishery products for human consumption. Aquatic products are a main source of animal proteins for the global population [1]. The use of chemicals such as antibiotics, probiotics, and other feed additives is essential for improving the productivity and commerciality of aquaculture [2,3]. Veterinary drugs such as fluoroquinolone and penicillin are widely used to prevent diseases in aquaculture. Anthelminitic drugs, such as benzimidazole, are mainly used to treat parasitic infections. β-Lactam antibiotics such as penicillin and cephalosporin are used as growth promoters and to treat bacterial infections such as respiratory or skin infections [4,5,6,7]. However, the intensive use of antibiotics in aquaculture can affect microbial populations in the aquatic environment and further promote the spread of drug-resistant bacteria and resistance genes [8,9]. These changes are important to human health because environmental microorganisms are a source of various genes that have transformed into virulence factors for acquisition by many human pathogens. In addition, several drugs, such as penicillin and quinolone, are classified as critically important for human medicine [10]. Thus, excessive use of these medicines should be reduced in animal products.

Previous monitoring studies report that a variety of veterinary drugs have been frequently detected in aquaculture animals in Korea. Fluoroquinolones were detected in 7.5% of 268 freshwater and seawater fish samples in 2011 [11]. Sulfadiazine, erythromycin, and trimethoprim have been commonly detected in aquaculture environments including in fish, sediments, and water in 2016 [12]. In addition, our previous research revealed a total detection rate of 22.7% (detected in 217 of the 958 samples) between 2014 and 2015 [13]. Therefore, a multi-residue analytical method is needed to evaluate residual levels of veterinary drugs in fishery products.

Many countries and institutions have adopted multi-residue and multi-class analysis to determine veterinary drug residues in animal products [14,15,16,17]. However, most previous studies focused on the residue analysis in livestock products. In this work, the sample preparation procedures were compared and evaluated to optimize the multi-residue determination of veterinary drugs in fish according to CODEX guidelines (CAC/GL-71) [18]. Finally, we optimized the analytical method for 60 target compounds using liquid chromatography-tandem mass spectrometry (LC-MS/MS). In addition, monitoring of real samples was performed to determine the residue levels of veterinary drugs in domestic fishery products. The proposed method is simple, with fast sample preparations and reliable recoveries to quantify and confirm the veterinary drug residues in fishery products.

## 2. Result and Discussion

### 2.1. LC-MS/MS Analysis

This study was conducted to develop a quantitative analytical method for multi-class veterinary drugs in fishery products. In the first step of method development, we tuned the mass spectrometer MS parameters for multiple reaction monitoring (MRM). MS parameters such as the electrospray source, the desolvation temperature, the con gas flow, the source temperature, and the capillary voltage have been shown to affect the signal intensity of all standards. To optimize the MS parameters, each standard solution was directly injected at a concentration of 50 mg/L in formic acid/methanol/water (1/499/500, *v*/*v*/*v*) into the mass spectrometer [19]. The MS parameters were optimized based on the mass spectra of all compounds. Protonated ([M+H]^+^) molecular ions were chosen as precursor ions of compounds based on their chemical properties in electrospray ionization (ESI). The exception was nitroxynil, for which the deprotonated ([M−H]^−^) molecular ion was used in a negative electrospray mode. The product ions were obtained by adjusting the cone voltage and the collision energy from the precursor ion. The most abundant transition from the precursor ions was used for quantification, whereas other transitions were used for confirmation. The scheduled MRM for each compound is listed in Table 1.

Chromatographic separation is important for identifying multi-class compounds. Preliminary trials were carried out to optimize the LC system conditions in a reverse phase X-SELECT C_18_ (2.1 mm × 150 mm × 3.5 µm). Columns filled with a C_18_ sorbent are widely used in the veterinary field for drug analysis in livestock and in fishery products [20]. Several mobile phases were tested to achieve a high-sensitivity detection of the analytes, which were (1) 0.1% formic acid and 5 mM ammonium formate in water/0.1% formic acid in methanol, (2) 0.1% formic acid and 5 mM ammonium formate in water/0.1% formic acid in acetonitrile, and (3) 0.1% formic acid in water/0.1% formic acid in acetonitrile. Methanol tends to interfere with the reliable analysis of some compounds such as β-lactam and penicillin due to the potential degradation [21]. Ammonium formate increases the ionic strength of the mobile phase or leads to the suppression of ionization, affecting the sensitivity of the analysis [22]. The combination of ammonium formate and formic acid has caused some compounds to show a narrower peak in the chromatograms [23]. However, there was no significant difference compared to using only formic acid to determine multiple residue drugs. Thus, the mobile phase (3) of 0.1% formic acid in water/0.1% formic acid in acetonitrile was used based on the peak shape, area, and stability and column wash time. Optimal gradient conditions were established for selected mobile phases to accurately separate target compounds within 12 min with high repeatability.

### 2.2. Comparison of Sample Preparation Methods

Methods of preparing samples have been developed for analyzing a wide range of veterinary drug residues [13,20,22,23,24,25]. In this study, we reviewed three methods of sample preparation (Table 2): Ministry of Food and Drug Safety in Korea (Method 1), Food and Environment Research Agency in United Kingdom (Method 2), and Food Safety and Inspection Service in United States (Method 3). Recovery tests in the flatfish tissue were performed to compare the three methods according to CODEX guidelines (70–120%). In Method 1, the extraction step was performed using EDTA as a chelating agent to improve the extraction recovery and prevent rapid chelation with metal ions [26,27]. A combination of formic acid and ammonium formate was used to improve peptide separation in the samples [13]. In Method 2, acidified acetonitrile was used as an extraction agent to eliminate interference from the matrix. Acidified acetonitrile is widely used to extract veterinary drugs from animal tissues [28,29]. Turnipseed et al. (2016) suggested that formic acid (0.2–1%) in acetonitrile may affect the degradation of several β-lactams. Formic acid in water causes a rapid degradation of monobasic penicillins. Thus, acetic acid was used to increase the acidity of the acetonitrile extractant [25]. In Method 3, water/acetonitrile solution was used for extraction because many compounds with different chemical groups and different physicochemical properties were present in the mixtures [30,31]. In addition, acetonitrile is typically preferred for precipitating proteins in tissue [25].

The extracted solutions in each method were purified using *n*-hexane, primary secondary amine (PSA), and octadecylsilane (C_18_). Animal tissues are rich in fats, lipids, and amino acids. Lipids can interfere with the analysis of some substances in animal tissues and contaminate the HPLC column [32]. Fats (specifically phospholipids) have been shown to cause significant matrix effects on ESI in the APCI MS analysis [33]. *n*-Hexane was added to eliminate some residual interference without the loss of target compounds. PSA and C_18_ absorbents have been used to prevent the co-extraction of interference compounds. The amino groups on the PSA can form strong hydrogen bonds with carboxylic acids and other polar organic acids [34,35]. C_18_ has been reported to allow the removal of oil and pigments [36]. 

**Figure 1:**
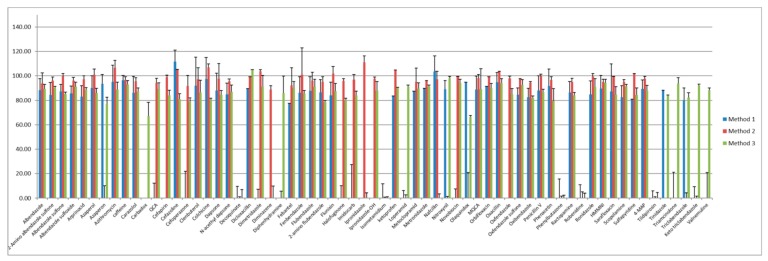
The comparison of targeted compounds for average recoveries (%) in flatfish in three multi-residue methods. Method 1: Ministry of Food and Drug Safety in Korea; Method 2: Food and Environment Research Agency in United Kingdom; Method 3: Food Safety and Inspection Service in United States.

Comparison of the three methods showed that Method 3 exhibited the highest recovery of 88% of the target compounds (Figure 1). In our previous method (Method 1), the recovery was below 50% for 22 analytes in fish samples. In addition, 50 compounds were affected by substances that interfered with the matrix. The linearity of the seven compounds was below the CODEX guidelines (*r*^2^ > 0.98). For Method 2, 19 compounds were not properly validated because of their poor peak shapes and high relative standard variations in fish samples. The validation results of Method 3 showed that only 8 compounds were incorrectly identified. Another sample preparation step may be required to simultaneously analyze 7 compounds (decoquinate, diminazene, novobiocin, phenylbutazone, robenidine, triclabendazole, and keto triclabendazole).

Based on the results obtained by comparison of the three methods, we selected and optimized Method 3. Briefly, acetonitrile/water solvents were used as the extract solution. A clean-up step was carried out by adding C_18_ and *n*-hexane. A concentration step was performed to improve the signal intensity of the compounds. A combination of methanol and water was used for residue analysis [24,25]. We evaporated the extract by placing the samples in a 40 °C water bath and dissolved the final residue in methanol/water (1/1, *v*/*v*). The temperature was controlled to maintain the target compounds under stable conditions.

### 2.3. Method Validation

Sixty-five compounds were initially selected for analysis based on the regulation of fishery products in Korea. Except for seven analytes, 60 compounds (47 drugs and their metabolites) were validated in the fishery products. The proposed method was evaluated according to selectivity, specificity, and linearity proposed by the CODEX guidelines. Selectivity was measured using blank samples of the spiked target compounds in fishery products. The chromatograms of the spiked sample solution and the standard mixture solution of target analytes were compared. The chromatograms of target compounds are shown in Figure S1. Specificity was evaluated using non-spiked blank samples at the same retention time for each analyte. Good linearity was observed with the regression coefficients (*r*^2^) of ≥ 0.98 for all compounds. Our results revealed good linearity of the targets within the target concentrations. Recovery was tested five times at three different concentrations (0.005 mg/kg, 0.01 mg/kg, and 0.02 mg/kg) in the fishery product samples. The recovery values were found to be between 73.2% and 115% and the coefficient variation (CV) ranged from 1.6% to 22.1%, meeting the guidelines. The recovery and CV at the target testing levels have been summarized in Table 3. The limit of detection (LOD) and the limit of quantification (LOQ) were calculated based on the signal to noise ratio (S/N) of the target compounds; S/N was ≥ 3 and ≥ 10 for LOD and LOQ, respectively. The matrix effects and LOQ values are presented in Table 4. In addition, inter-laboratory validation was conducted by three different institutions. As a result, the linearity of the calibration curve showed an *r*^2^ ≥ 0.98. Recovery of the analytes ranged from 64.3% to 115% and repeatability ranged from 1.1% to 22.2%. The results of inter-lab validation demonstrate the validity of the proposed method.

### 2.4. Matrix Effect

The samples were analyzed by LC-MS/MS. The sample matrix has been reported to affect quantification of the target analytes because of ionization suppression or enhancement for the analyte/matrix combination [28]. The main sources of these effects were endogenous substances such as ionic species (salt) and various organic molecules (lipid, peptide, and metabolites with a chemical structure close to the target analyte structure) [37]. In this study, the matrix-matched calibration curves were applied to adjust the matrix effects. The matrix-matched and the solvent standard curve were compared to evaluate the matrix effects (ME) which were calculated as follows:

(1) $$\mathrm{ME}\left(\%\right)=\left(\frac{Slope_{matrix\;matched\;standard\;curve}}{Slope_{solvent\;standard\;curve}}-1\right)\times 100$$

A matrix effect enhances the ionization efficiency of the target compounds, whereas a negative effect indicates the suppression of ionization. As shown in Table 4, ionization suppression occurred owing to the matrix effects for most of the target drugs. Ion enhancement of the signal was observed for several compounds (azithromycin, cefapirin, desacetylcefapirin, cefoperazone, imidocarb, and tildipirosin) in the fish samples including eel and shrimp (data was not shown). Gbylik et al. (2013) obtained similar results, although different multi-residue analysis was performed [38]. Tildipirosin is a high-signal polar compound that has been shown to be affected more by the sample matrix effects than by non-polar molecules [39]. Dickson et al. (2014) showed that tildipirosin had slightly larger matrix effects than other macrolide compounds because it co-eluted with other polar compounds [40]. Therefore, in the case of tildipirosin, analytical methods using an internal standard may be useful for correcting analytical differences during sample analysis.

Although d-SPE treatment was used to reduce the matrix effect, a negative matrix effect was observed in the fish samples. The matrix effect can be major issue for the development of a multi-residue method using LC-MS/MS. Therefore, optimizing sample preparation and dilution procedures, and manipulating LC and MS conditions, are needed to reduce the matrix effect [41]. Isotope-labeled internal standards also can be alternative method to significantly reduce the matrix effect [42]. Further investigation is required for optimization of simultaneous determination of veterinary drug residues in fish matrices.

### 2.5. Application to Real Samples

The fishery product samples (*n* = 102) were collected and monitored to evaluate veterinary drug residues using the proposed method. Seven kinds of fishery products were collected from a domestic market in 2019. The fishery product samples were analyzed and checked to ensure the retention time, quantification ion, confirmation ion, and ion ratio were consistent with standards. The detected sample did not exceed the LOQ and Korean maximum residue limits. Tildipirosin was detected at 0.001 mg/kg in one sample of catfish. Further studies are needed to control the veterinary drug residues in many samples of fishery products using the proposed method.

## 3. Materials and Methods

### 3.1. Chemicals, Materials, and Solution

The chemical standards (Table S1) of veterinary drugs were purchased from Dr. Ehrenstofer (Augsburg, Germany), Wako Pure Chemical Industries Inc. (Osaka, Japan), TRC (Toronto, Canada), USP (Rockville, MD, USA), and Sigma (St. Louis, MO, USA). Acetonitrile, methanol, and *n*-hexane were purchased from Merck Inc. (Darmstadt, Germany) as HPLC gradations. Formic acid was purchased from Sigma and C_18_ (55–105 μm, 125 Å) was purchased from Waters (Milford, MA, USA). PSA was purchased form Agilent technologies (Santa Clara, CA, USA). All glass apparatus used in the experiment was cleaned and dried with cleaning solution, methanol, and tertiary distilled water (Arium 61316 composing Arium 611VF). Sartorius (Gottingen, Germany) and centrifuge tube of Corning (NY, USA) were used. The filter was purchased and used with a size of 0.2 μm polytetrafluoroethylene (PTFE) material from Teknokroma (Barcellona, Spain). For the mixed extraction of the sample, we used a mixer (MMV-1000W, Eyela, Tokyo, Japan) and the centrifuge (HERAEUSE Megafuge 16R, ThermoFisher Scientific, Waltham, MA, USA). The standard stock solutions were prepared for each compound in methanol, 50% methanol in water (*v*/*v*) and dimethyl sulfoxide (100 mg/L). All working solution mixtures of 1 µg/mL were diluted in 50% methanol in water (*v*/*v*). Standard solutions and working solutions were stored at −20 °C.

### 3.2. LC-MS/MS Analysis

The instrumental analysis was performed using ultra-performance liquid chromatography (xevo TQ-S) with X-SELECT C_18_ (2.1 mm × 150 mm × 3.5 µm) column by Waters (Milford, MA, USA). The mobile phase was 0.1 % formic acid in water (A) and 0.1 % formic acid in acetonitrile (B). The gradient mode was follow; (from min 0: 5% B, min 0.5–5.5: 60% B, min 5.5–6.0: 100% B, min 6.0–10.0: 100% B, min 10.0–10.2: 5% B, min 10.2–12.0: 5% B) at flow rate of 0.3 mL min^−1^. The injection volume was 5 µL. Mass parameters include capillary voltage 3.6 kV (ESI+) and −2.8 kV (ESI−). The source and desolvation temperatures were set at 150 °C and 500 °C, respectively, and desolvation gas (nitrogen) flow rate was 600 L h^-1^. The column and auto sampler temperature were maintained at 40 °C and 15 °C, respectively. The collision argon gas was set at a pressure of 4 × 10^−3^ mbar. Triple quadrupole tandem mass analysis was performed in positive (ESI+) and negative (ESI−) mode. Data collection was performed in MRM mode using MassLynx software (Waters, UK).

### 3.3. Sample Preparation

Flatfish samples were purchased from a market in the Republic of Korea. They were homogenized and stored in the freezer at −20 °C until use in the experiment. Two grams of the homogenized sample was weighed and placed into a 50 mL centrifuge tube. Ten milliliters of acetonitrile/water (4/1, *v*/*v*) was added and mixed with the sample for 5 minutes. The tube with the sample was centrifuged at 4500× *g* and 4 °C for 10 min. The centrifuged extract was acquired and transferred to a new centrifuge tube with 500 mg C_18_ powder. Then, 10 mL of acetonitrile saturated hexane was added to the tube and shaken for 1 minutes. After centrifuging for 5 minutes at 4500× *g* and 4 °C, 5 mL of the bottom solution below the hexane layer was transferred to a new centrifuge tube. Subsequently, the extraction solution (5 mL) were evaporated at 40 °C under N_2_ gas. The residue was then dissolved with 1 mL of methanol/water (1/1, *v*/*v*), filtered through 0.2 μm PTFE membrane filter, and placed in a pp-vial.

### 3.4. Method Validation

The proposed method was validated according to the procedures described in the Codex Alimentarius Commission guidelines (CAC/GL-71) [17]. In terms of selectivity, linearity, accuracy, precision, LOD, and LOQ, the results for the method were obtained. Accuracy and precision were expressed as recovery and CV. The target concentration (TC) was set at 0.01 mg/kg, and validation was performed for concentration level 0.5 × TC, 1 × TC, and 2 × TC. The calibration curves were obtained by matrix-matched standard solution at six points (0.0025, 0.005, 0.01, 0.015, 0.02, and 0.03 mg/kg). The validation was conducted by spiking blank fish with a mixed working solution at three concentration levels. Recovery and CV were obtained through five repeated experiments. The LODs and LOQs were defined with signal-to-noise ratios (S/N ratio) ≥3 and ≥10, respectively.

## 4. Conclusions

The quantitative multi-residue determination of 60 veterinary drugs in fish tissue by LC-MS/MS as developed. Three methods of sample preparation were compared. The extraction solvent of a mixed solution of water and acetonitrile was selected to dissolve various substances. C_18_ and hexane were used to remove interfering substance in flatfish. This multi-residue method showed clear sample cleanup efficiency and demonstrated satisfactory recovery, accuracy, and precision for 60 veterinary drugs in flatfish samples. The proposed method can be applied to the monitoring of real samples. This study may be used to determine the concentration of veterinary drug residues in fishery products.

## Figures and Tables

**Table 1:** LC-MS/MS parameters of target veterinary drugs.

Class	Compounds	ESI (+/−)	Molecular Weight	Precursor Ion (*m*/*z*)	Product ion (*m*/*z*)	Collision Energy (eV)	Cone Voltage	Retention Time
Benzimidazoles	Albendazole	+	265.3	266.2	163	35	30	5.31
					191	25		
					**234**	15		
	2-Amino albendazole sulfone	+	239.3	240.1	79	42	30	3.25
					105	38		
					**198**	18		
	Albendazole sulfone	+	297.3	298.1	**159**	30	30	4.57
					224	25		
					266	13		
	Albendazole sulfoxide	+	281.3	282.1	159	35	30	3.81
					208	25		
					**240**	13		
	Febantel	+	446.5	447.3	280	28	20	7.17
					**383**	16		
					415	10		
	Fenbendazole	+	299.3	300.3	131	42	40	6.07
					159	34		
					**268**	18		
	Flubendazole	+	313.3	314.3	95	42	50	5.56
					123	32		
					**282**	18		
	2-Amino flubendazole	+	255.3	256.1	95	35	30	4.25
					**123**	28		
					133	35		
	Oxfendazole	+	315.3	316.1	**159**	30	30	4.47
					191	15		
					284	15		
	Oxfendazole sulfone	+	331.3	332.1	131	40	30	5.26
					159	32		
					**300**	20		
	Oxibendazole	+	249.3	250.3	148	15	65	4.49
					176	15		
					**218**	10		
Cefalosporines	Cefapirin	+	423.5	424.0	152	20	30	2.92
					181	20		
					**292**	13		
	Desacetylcefapirin	+	403.4	382.2	**112**	20	35	2.13
					152	25		
					193	25		
	Cefazoline	+	454.5	455.1	**155**	15	30	3.87
					322	9		
	Cefoperazone	+	645.7	646.1	**143**	30	22	4.27
					290	22		
					530	10		
Coccidiostats	Halofuginone	+	414.7	414.2	**100**	18	20	4.37
					120	16		
					138	18		
Macrolides	Azithromycin	+	748.9	746.5	116	40	30	3.95
					158	40		
					**592**	25		
	Tildipirosin	+	734.0	734.5	**98**	35	30	2.91
					156	35		
					174	35		
Nitroimidazoles	Dimetridazole	+	141.1	142.0	81	18	30	3.39
					**95**	10		
					96	10		
	Ipronidazole	+	169.1	170	96	20	30	5.06
					**109**	20		
					124	20		
	Ipronidazole-OH	+	185.1	186.2	82	20	28	4.17
					107	26		
					**122**	16		
	Metronidazole	+	171.2	172.1	56	15	30	3.00
					82	20		
					**128**	10		
	Metronidazole-OH	+	187.1	187.9	68	18	28	2.73
					**123**	12		
					126	15		
	Tinidazole	+	247.3	248.1	93	18	30	3.87
					**121**	15		
					128	18		
	Ronidazole	+	200.2	201.0	55	18	30	3.33
					110	15		
					**140**	8		
	HMMNI (2-hydroxymethyl-1-methyl-5-nitroimidazole)	+	157.1	157.9	55	15	30	3.05
					94	20		
					**140**	12		
Penicillins	Dicloxacillin	+	470.3	470.0	**160**	15	30	6.72
					200	30		
					311	15		
	Nafcillin	+	414.5	415.4	115	55	30	6.46
					171	35		
					**199**	35		
	Oxacillin	+	401.4	402.0	114	20	30	6.04
					144	12		
					**160**	12		
	Penicillin V	+	350.4	351.2	160	15	65	5.73
					**229**	15		
					257	10		
	4-MAP (4-methylamino antipyrine)	+	217.3	218.2	**97**	15	35	2.91
					125	12		
					187	12		
Quinolones	Sarafloxacin	+	385.4	386.1	**299**	25	30	3.88
					342	15		
					368	18		
	Orbifloxacin	+	395.4	396.1	267	35	30	3.72
					**295**	25		
					352	15		
Quinoxalines	Carbadox	+	262.2	263.1	103	30	30	3.54
					129	30		
					**231**	10		
	QCA (Quinoxaline-2-carboxylic acid)	+	174.2	175.0	104	20	30	3.89
					**129**	10		
					131	10		
	Olaquindox	+	263.3	264.1	**143**	30	40	2.77
					212	20		
					221	15		
	MQCA (3-methylquinoxaline-2- carboxylic acid)	+	188.2	189.1	92	22	32	4.10
					118	20		
					**145**	12		
Sulfonamides	Dapsone	+	248.3	249.1	92	20	30	4.38
					108	20		
					**156**	15		
	N-acethyl dapsone	+	290.3	291.1	92	20	30	4.54
					108	20		
					**156**	15		
	Sulfapyridine	+	249.3	250.1	92	28	30	3.59
					108	22		
					**156**	15		
Tranquillisers	Arprinocid	+	277.7	278.3	83	46	40	4.22
					107	46		
					**143**	28		
	Azaperol	+	329.4	330.3	109	45	30	3.49
					**121**	20		
					149	25		
	Azaperon	+	327.4	328.2	123	25	30	3.76
					147	20		
					**165**	20		
	Carazolol	+	298.4	299.5	**116**	15	30	4.36
					185	20		
					196	20		
Other	Caffeine	+	194.2	195.2	42	25	30	3.34
					110	20		
					**138**	15		
	Clenbuterol	+	277.2	277.1	132	25	30	3.98
					168	30		
					**203**	15		
	Colchicine	+	399.4	400.2	282	28	30	4.79
					**310**	25		
					358	20		
	Diphenhydramine	+	355.4	356.3	128	46	30	4.93
					152	30		
					**167**	10		
	Flunixin	+	296.2	297.1	210	30	30	6.90
					264	35		
					**279**	25		
	Imidocarb	+	348.4	349.3	145	46	20	2.69
					162	22		
					**188**	25		
	Isometamidium	+	460.6	460.2	269	45	30	3.87
					**298**	25		
					313	30		
	Ketoprofen	+	254.3	255.1	77	34	42	6.55
					**105**	22		
					194	24		
	Loperamide	+	477.0	477.6	210	45	30	5.95
					238	45		
					**266**	25		
	Metoclopramide	+	299.8	300.2	140	40	30	3.73
					182	30		
					**226**	15		
	Nitroxynil	−	290.0	288.8	116	34	66	6.41
					127	22		
					**162**	20		
	Phenacetin	+	179.2	180.2	**110**	15	30	5.02
					138	12		
					152	12		
	Ractopamine	+	301.1	302.2	107	25	30	3.56
					121	20		
					**164**	15		
	Scopolamine	+	303.4	304.2	103	32	30	3.29
					**138**	20		
					156	15		
	Triamcinolone	+	394.4	395.1	339	10	30	4.49
					357	10		
					**375**	8		
	Valnemuline	+	564.8	565.4	72	35	30	5.58
					164	32		
					**263**	15		

^a^ Product ion in bold indicate quantitative ions.

**Table 2:** Comparison of flow chart for three methods.

Methods	1 (MFDS)	2 (FERA)	3 (FSIS)
Sample	2 g of samples		
Extraction	0.1 M EDTA in 50 mM ammonium acetate (pH 4.0) (1 mL)	1 % Acetic acid in water (1 mL)	Water/Acetonitrile (1/4, *v*/*v*) (10 mL)
	2 mM Ammonium formate in water/ACN (1/4, *v*/*v*) (9 mL)	Acetonitrile (10 mL)	
Purification	C_18_ (250 mg)	Na_2_SO_4_ (2 g)	C_18_ (500 mg)
		Hexane (10 mL)	
	PSA (250 mg)	C_18_ (100 mg)	Acetonitrile saturated hexane (10 mL)
		PSA (100 mg)	
Evaporation	N_2_(*g*), 40 °C		
Reconstitution	Methanol/Water (1/1, *v*/*v*) (1 mL)		
Filter	PVDF	PTFE	PTFE
Analysis	LC-MS/MS		

**Table 3:** Recovery and CV (coefficient variation) at target testing levels in flatfish.

Compounds	Target Testing Level (mg/kg)	Flatfish (*n* = 5)		Compounds	Target Testing Level (mg/kg)	Flatfish (*n* = 5)	
		Recovery (%)	CV (%)			Recovery (%)	CV (%)
Albendazole	0.005	96.5	4.7	Ipronidazole	0.005	106	10.5
	0.01	94.1	5.2		0.01	102	19.5
	0.02	92.7	4.2		0.02	103	9.8
2-Amino albendazole sulfone	0.005	96.5	3.3	Ipronidazole-OH	0.005	81.5	3.3
	0.01	95.4	5.2		0.01	94.7	11.1
	0.02	94.5	4.6		0.02	94.9	5.6
Albendazole sulfone	0.005	98.7	5.4	Isometamidium	0.005	97.7	4.0
	0.01	98.5	3.8		0.01	89.1	10.5
	0.02	95.6	2.2		0.02	73.2	16.4
Albendazole sulfoxide	0.005	92.8	3.7	ketoprofen	0.005	98.1	6.4
	0.01	95.5	4.0		0.01	101	7.5
	0.02	94.1	2.8		0.02	97.8	5.6
Arprinocid	0.005	93.7	2.3	Loperamide	0.005	108	5.5
	0.01	96.1	3.4		0.01	95.2	11.1
	0.02	95.2	2.9		0.02	83.0	13.1
Azaperon	0.005	81.8	9.6	Metoclopramide	0.005	95.6	1.6
	0.01	87.4	17.2		0.01	97.9	4.4
	0.02	90.9	8.1		0.02	94.7	4.8
Azaperol	0.005	86.9	6.0	Metronidazole	0.005	104	3.8
	0.01	94.0	9.6		0.01	100	6.4
	0.02	96.6	5.9		0.02	96.0	4.4
Azithromycin	0.005	82.9	4.8	Metronidazole-OH	0.005	112	3.2
	0.01	83.9	3.9		0.01	105	5.4
	0.02	80.5	5.1		0.02	102	3.8
Caffeine	0.005	113	12.4	Nafcillin	0.005	107	6.8
	0.01	99.6	6.8		0.01	102	9.9
	0.02	95.8	5.5		0.02	97.1	7.2
Carazolol	0.005	96.4	5.6	Nitroxynil	0.005	88.6	3.0
	0.01	94.5	6.3		0.01	103	4.7
	0.02	90.6	6.1		0.02	98.2	1.8
Carbadox	0.005	102	22.1	Olaquindox	0.005	106	10.6
	0.01	111	21.4		0.01	102	16.6
	0.02	96.7	21.1		0.02	104	6.1
QCA	0.005	115	12.6	MQCA	0.005	103	4.5
	0.01	108	5.8		0.01	96.7	8.1
	0.02	94.4	2.9		0.02	90.7	6.2
Cefapirin	0.005	107	3.3	Orbifloxacin	0.005	105	10.1
	0.01	93.5	5.0		0.01	96.3	6.6
	0.02	85.0	3.3		0.02	91.7	4.1
Desacetylcefapirin	0.005	110	3.0	Oxacillin	0.005	103	8.9
	0.01	105	5.6		0.01	97.5	11.8
	0.02	104	3.9		0.02	94.5	7.7
Cefazoline	0.005	100	9.4	Oxfendazole	0.005	101	3.7
	0.01	98.9	9.3		0.01	100	3.8
	0.02	100	5.0		0.02	97.4	3.0
Cefoperazone	0.005	106	6.2	Oxfendazole sulfone	0.005	94.5	2.6
	0.01	95.7	7.9		0.01	98.6	2.0
	0.02	90.9	3.9		0.02	97.6	1.6
Clenbuterol	0.005	93.9	3.7	Oxibendazole	0.005	93.7	5.0
	0.01	96.0	5.9		0.01	93.4	4.8
	0.02	92.6	4.2		0.02	90.7	4.6
Colchicine	0.005	95.8	3.3	Penicillin V	0.005	96.2	8.6
	0.01	94.2	4.5		0.01	91.4	5.7
	0.02	93.2	4.1		0.02	89.2	5.6
Dapsone	0.005	98.6	3.8	Phenacetin	0.005	101	4.9
	0.01	96.9	3.9		0.01	102	6.2
	0.02	94.4	3.1		0.02	106	11.0
N-acethyl dapsone	0.005	97.7	4.2	Ractopamine	0.005	97.2	2.0
	0.01	96.9	4.3		0.01	96.5	5.8
	0.02	94.0	4.6		0.02	95.2	4.1
Dicloxacillin	0.005	111	15.3	Ronidazole	0.005	97.2	8.8
	0.01	99.9	18.1		0.01	94.8	8.0
	0.02	95.1	15.9		0.02	90.2	5.3
Dimetridazole	0.005	87.4	9.6	HMMNI	0.005	103	3.7
	0.01	89.3	14.7		0.01	96.9	5.6
	0.02	86.6	8.1		0.02	96.2	5.0
Diphenhydramine	0.005	89.4	11.6	Sarafloxacin	0.005	105	2.4
	0.01	96.0	13.6		0.01	98.5	4.2
	0.02	86.6	16.0		0.02	97.5	5.9
Febantel	0.005	102	15.8	Scopolamine	0.005	95.7	3.8
	0.01	102	15.5		0.01	94.8	4.8
	0.02	104	12.7		0.02	94.2	6.0
Fenbendazole	0.005	96.7	5.0	Sulfapyridine	0.005	99.8	2.8
	0.01	94.2	8.5		0.01	96.1	5.0
	0.02	89.6	6.5		0.02	93.7	5.1
Flubendazole	0.005	92.9	4.4	4-MAP	0.005	105	4.0
	0.01	94.5	4.0		0.01	96.6	9.0
	0.02	93.8	2.8		0.02	93.9	6.7
2-Amino flubendazole	0.005	102	3.7	Tildipirosin	0.005	97.8	5.1
	0.01	93.8	5.4		0.01	106	10.5
	0.02	88.3	8.1		0.02	97.2	11.9
Flunixin	0.005	98.8	7.4	Tinidazole	0.005	100	3.9
	0.01	103	7.9		0.01	99.6	5.2
	0.02	104	5.1		0.02	98.1	5.0
Halofuginone	0.005	103	5.5	Triamcinolone	0.005	93.8	14.7
	0.01	96.9	7.5		0.01	95.7	10.5
	0.02	92.4	9.8		0.02	88.9	6.6
Imidocarb	0.005	102	1.7	Valnemuline	0.005	107	8.8
	0.01	97.9	14.6		0.01	95.9	13.8
	0.02	100	14.4		0.02	86.5	10.1

**Table 4:** Matrix effects (%) and limit of quantification (LOQ) in flatfish.

Compounds	Matrix Effect (%)	LOQ (mg/kg)	Compounds	Matrix Effect (%)	LOQ (mg/kg)
Albendazole	−45	0.0005	Ipronidazole	−78	0.0005
2-Amino albendazole sulfone	−40	0.0010	Ipronidazole-OH	−39	0.0050
Albendazole sulfone	−38	0.0010	Isometamidium	−56	0.0030
Albendazole sulfoxide	−37	0.0020	ketoprofen	−44	0.0020
Arprinocid	−40	0.0005	Loperamide	−46	0.0015
Azaperon	−70	0.0010	Metoclopramide	−45	0.0005
Azaperol	−78	0.0010	Metronidazole	−49	0.0030
Azithromycin	−12	0.0005	Metronidazole-OH	−58	0.0040
caffeine	−40	0.0020	Nafcillin	−43	0.0014
Carazolol	−53	0.0006	Nitroxynil	−3	0.0030
Carbadox	−74	0.0050	Olaquindox	−74	0.0050
QCA	−34	0.0050	MQCA	−49	0.0050
Cefapirin	−15	0.0040	Orbifloxacin	−47	0.0010
Desacetylcefapirin	5	0.0015	Oxacillin	−45	0.0010
Cefazoline	−27	0.0020	Oxfendazole	−38	0.0010
Cefoperazone	−28	0.0050	Oxfendazole sulfone	−33	0.0010
Clenbuterol	−52	0.0010	Oxibendazole	−46	0.0005
Colchicine	−31	0.0020	Penicillin V	−38	0.0010
Dapson	−43	0.0020	Phenacetin	−46	0.0010
N-acethyl dapsone	−40	0.0010	Ractopamine	−51	0.0005
Dicloxacillin	−58	0.0005	Ronidazole	−47	0.0040
Dimetridazole	−54	0.0050	HMMNI	−57	0.0050
Diphenhydramine	−70	0.0010	Sarafloxacin	−24	0.0025
Febantel	−73	0.0020	pbScopolamine	−56	0.0020
Fenbendazole	−43	0.0005	Sulfapyridine	−44	0.0020
Flubendazole	−33	0.0005	4-MAP	−64	0.0020
2-Amino flubendazole	−44	0.0050	Tildipirosin	208	0.0020
Flunixin	−38	0.0007	Tinidazole	−46	0.0010
Halofuginone	−46	0.0010	Triamcinolone	−38	0.0050
Imidocarb	−70	0.0050	Valnemuline	−51	0.0005

## Supplementary Materials

The following are available online at https://www.mdpi.com/1420-3049/25/5/1206/s1, Figure S1: The chromatograms of veterinary drugs (at 0.01 mg/kg level), Table S1: Manufacturer of standards.
