# Supplementary material for: Comparison of Sample Preparation and Determination of 60 Veterinary Drug Residues in Flatfish Using Liquid Chromatography-Tandem Mass Spectrometry
Source: Molecules. 2020 Mar 7;25(5):1206. doi: 10.3390/molecules25051206 (PMC7179470; doi:10.3390/molecules25051206)
Supplement: Supplementary file 1 [file molecules-25-01206-s001.pdf]

# Supplementary Materials

# Comparison of sample preparations for determining the veterinary drug residues using liquid chromatography-tandem mass spectrometry in fishery products.

Joohye Kim[a], Hyunjin Park[a], Hui-Seung Kang*[a]

[a] *Pesticide and Veterinary Drug Residues Division, National Institute of Food and Drug Safety Evaluation, Osong, Chungcheongbuk-do 363-700, South Korea*

**Table S1.** Manufacturer of standards

| Compounds | Manufacturer | Compounds | Manufacturer |
|---|---|---|---|
| Albendazole | Fluka | 4-MAP (4-methylamino antipyrine) | Sigma-Aldrich |
| 2-Amino albendazole sulfone | Dr. Ehrenstorfer | Sarafloxacin | Dr. Ehrenstorfer |
| Albendazole sulfone | Sigma-Aldrich | Orbifloxacin | Dr. Ehrenstorfer |
| Albendazole sulfoxide | Sigma-Aldrich | Carbadox | Sigma-Aldrich |
| Febantel | Dr. Ehrenstorfer | QCA (Quinoxaline-2-carboxylic acid) | Sigma-Aldrich |
| Fenbendazole | Sigma-Aldrich | Olaquindox | Wako |
| Flubendazole | Dr. Ehrenstorfer | MQCA (3-methylquinoxaline-2-carboxylic acid) | Sigma-Aldrich |
| 2-amino flubendazole | Sigma-Aldrich | Dapsone | Dr. Ehrenstorfer |
| Oxfendazole | Dr. Ehrenstorfer | N-acethyl dapsone | TRC |
| Oxfendazole sulfone | Wako | Sulfapyridine | Sigma-Aldrich |
| Oxibendazole | Sigma-Aldrich | Arprinocid | TRC |
| Cefapirin | Fluka | Azaperol | Sigma-Aldrich |
| Desacetylcefapirin | TRC | Azaperon | Fluka |
| Cefazoline | USP | Carazolol | Dr. Ehrenstorfer |
| Cefoperazone | USP | Caffeine | Sigma-Aldrich |
| Halofuginone | Sigma-Aldrich | Clenbuterol | Dr. Ehrenstorfer |
| Azithromycin | USP | Colchicine | Sigma-Aldrich |
| Tildipirosin | TRC | Diphenhydramine | Sigma-Aldrich |
| Dimetridazole | Dr. Ehrenstorfer | Flunixin | Dr. Ehrenstorfer |
| Ipronidazole | Sigma-Aldrich | Imidocarb | Dr. Ehrenstorfer |
| Ipronidazole-OH | Sigma-Aldrich | Isometamidium | TRC |
| Metronidazole | Dr. Ehrenstorfer | ketoprofen | Dr. Ehrenstorfer |
| Metronidazole-OH | Dr. Ehrenstorfer | Loperamide | Fluka |
| Tinidazole | TRC | Metoclopramide | Sigma-Aldrich |
| Ronidazole | Dr. Ehrenstorfer | Nitroxynil | Dr. Ehrenstorfer |

| | | | |
|---|---|---|---|
| HMMNI (2-hydroxymethyl-1-methyl-5-nitroimidazole) | Fluka | Phenacetin | Dr. Ehrenstorfer |
| Dicloxacillin | Sigma-Aldrich | Ractopamine | Sigma-Aldrich |
| Nafcillin | USP | Scopolamine | Sigma-Aldrich |
| Oxacillin | USP | Triamcinolone | Sigma-Aldrich |
| Penicillin V | USP | Valnemuline | Sigma-Aldrich |

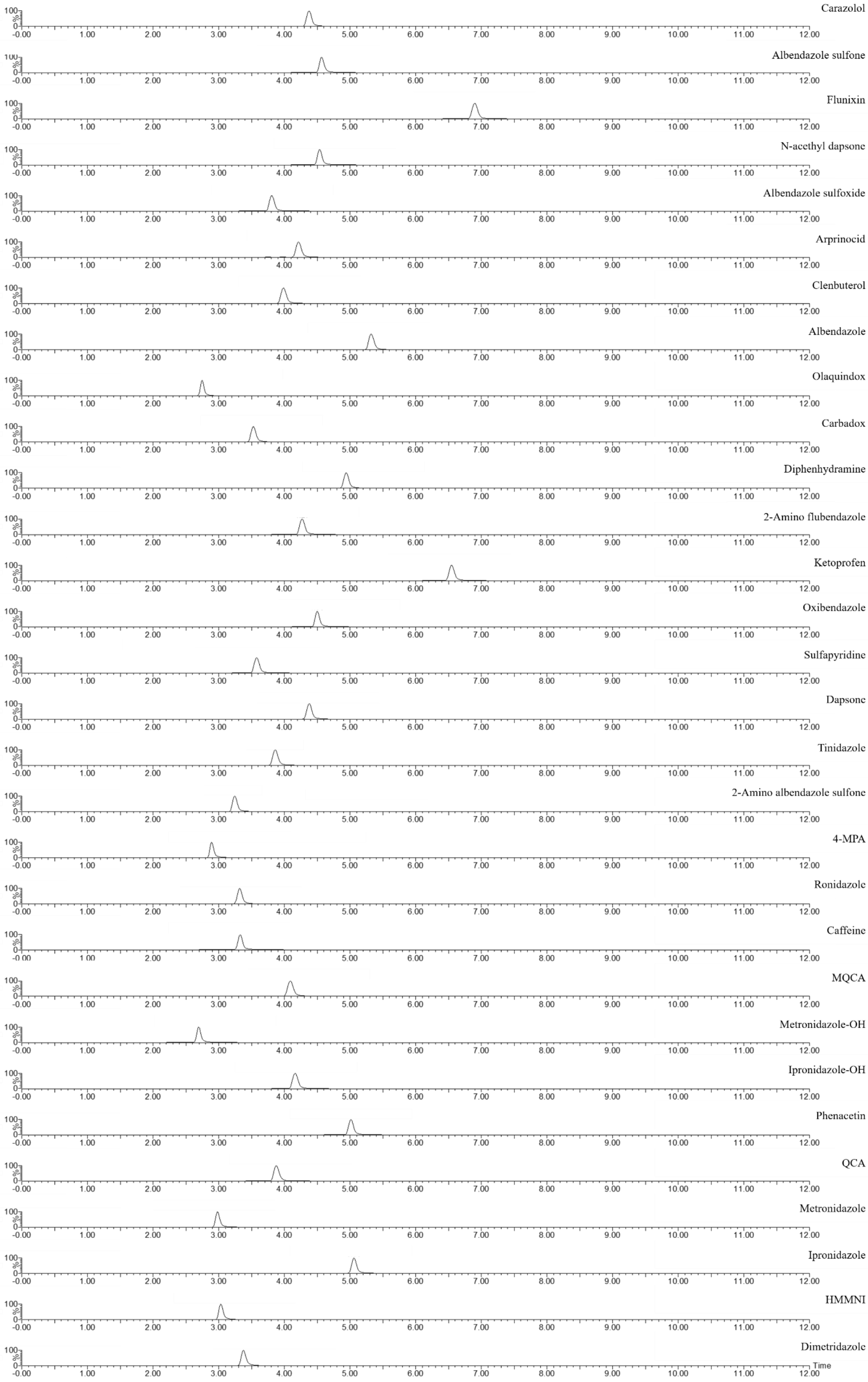
Carazolol
Albendazole sulfone
Flunixin
N-acethyl dapsone
Albendazole sulfoxide
Arprinocid
Clenbuterol
Albendazole
Olaquindox
Carbadox
Diphenhydramine
2-Amino flubendazole
Ketoprofen
Oxibendazole
Sulfapyridine
Dapsone
Tinidazole
2-Amino albendazole sulfone
4-MPA
Ronidazole
Caffeine
MQCA
Metronidazole-OH
Ipronidazole-OH
Phenacetin
QCA
Metronidazole
Ipronidazole
HMMNI
Dimetridazole
100
%
0
-0.00
1.00
2.00
3.00
4.00
5.00
6.00
7.00
8.00
9.00
10.00
11.00
12.00
Time

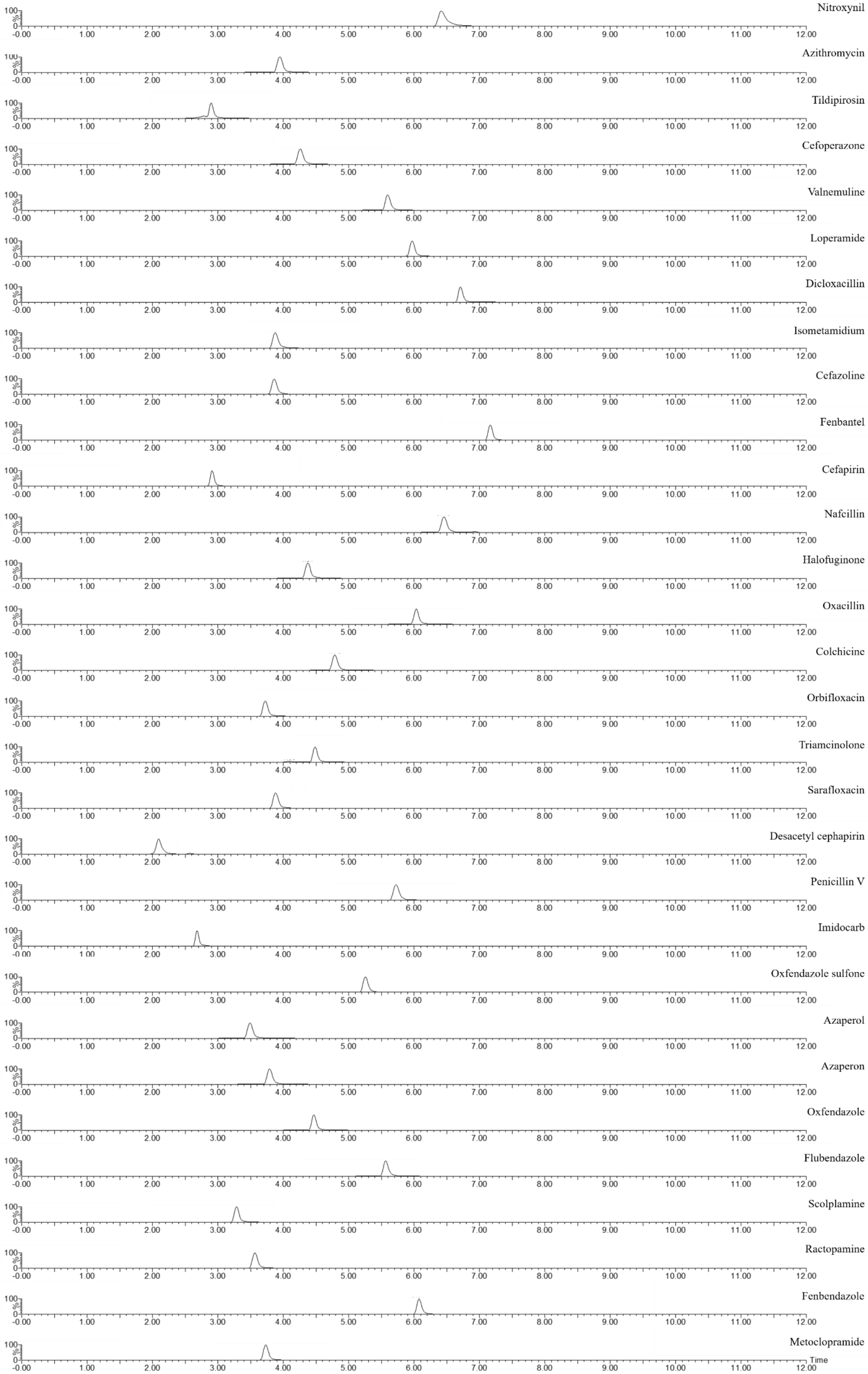
Nitroxynil
Azithromycin
Tildipirosin
Cefoperazone
Valnemuline
Loperamide
Dicloxacillin
Isometamidium
Cefazoline
Fenbantel
Cefapirin
Nafcillin
Halofuginone
Oxacillin
Colchicine
Orbifloxacin
Triamcinolone
Sarafloxacin
Desacetyl cephapirin
Penicillin V
Imidocarb
Oxfendazole sulfone
Azaperol
Azaperon
Oxfendazole
Flubendazole
Scolplamine
Ractopamine
Fenbendazole
Metoclopramide
100
%
0
-0.00
1.00
2.00
3.00
4.00
5.00
6.00
7.00
8.00
9.00
10.00
11.00
12.00
Time

**Figure S1**. The chromatograms of veterinary drugs (at 0.01 mg/kg level)
